# Literary Notes

**Published:** 1897-01

**Authors:** 


### Literary Notes.

Annales d'Oculistique.—An English edition of this venerable ophthalmic journal was announced in January, 1895. Its direction here was undertaken by Dr. Geo. T. Stevens, of New York, and received cordial professional support. Issues of the journal were continued from January, 1895, to July, 1896, inclusive. The subscribers are now informed by Dr. Stevens that such interpretations have been put upon the agreement by the managers at Paris as to make unity of action and interest impossible, and he is compelled to discontinue its publication here.

It is greatly to be regretted that such an excellent journal cannot be made truly international and continued as such.

---

Ophthalmic Record.—This journal, which has been published by Dr. G. C. Savage, of Nashville, Tenn., for five years past, has now been transferred to other hands, with headquarters at Chicago. It will be under the direction of an editorial board, of which Dr. T. A. Woodruff, of Chicago, will be editorial secretary. The size of the *Record* will be increased, and it will be otherwise improved. The first issue of the new series will be made early in January, 1897.

---

A Useful Publication.—Victor Koechl & Co., of New York, importers of valuable modern medicinal preparations, such as antipyrin, diphtheria antitoxin—"Behring," lanolin, dermatol, tuberculin and the like, are publishing a little monthly, entitled, *Therapeutic Progress*, devoted to brief reports and condensed abstracts pertaining to new remedies. Each issue contains something of practical interest to the physician. We have, during the past year, received a number of copies of this little paper, and can vouch for its usefulness and also for the able manner in which it is edited.

Any physician who desires to keep informed in regard to the newer remedies can receive *Therapeutic Progress*, free of charge, during 1897, by addressing a request to Victor Koechl & Co., 79 Murray street, New York.

---

WE TAKE pleasure in calling attention to a very handsome pamphlet, presenting some practical and interesting facts regarding tongaline and the different troubles for which that remedy is intended — namely, rheumatism, neuralgia, nervous headache, grip, gout, sciatica and lumbago. The brochure is embellished with original drawings and also handsome photogravures of a number of eminent members of the medical profession, now deceased, which render it attractive. It is the aim of the publishers to mail a copy to every physician in the country, but any who fail to receive such, can obtain one by applying to the Mellier Drug Company, St. Louis, mentioning the JOURNAL.

---

THE Antikamnia Chemical Company, of St. Louis, is sending to a number of its friends a unique Christmas gift that is also a useful souvenir. It is a calendar illustrated with skeleton sketches reproduced from original water colors painted by Dr. Louis Crusius, a noted artist-physician of St. Louis. The letter of transmittal truthfully says that the facial expression so faithfully depicted is original from an artistic view, and the artist's skill in giving life expressions to the skull is truly wonderful.

The enterprise and liberality of the Antikamnia Chemical Company are well known, and we beg to acknowledge the receipt of this Christmas souvenir and to express our thanks for the courtesy.

---

THE *Medical Mirror*, of St. Louis, announces in its December edition that a new company has been formed, under the title Love-Hadley Publishing Company, that will hereafter print the *Mirror*. The accomplished editor, Dr. I. N. Love, will thus be relieved of much detail work, and be able to devote more time to his editorial duties. Perhaps we should say more leisure, because it is a well-known fact that the editor of the *Mirror* is not "a slave to time." We congratulate the *Mirror* on its prosperity and the wonderful strides it has made in establishing itself in the hearts of its readers as well as in the esteem of its subscribers and advertisers.

The Columbia calendar for 1897, issued by the Pope Manufacturing Company, is one of the most useful desk calendars of which we have any knowledge. While we observe that one of its objects is to advertise the Columbia bicycles, it is by no means its only purpose. It teaches the joys of outdoor life and the comfort and economy of good roads, and on each day's slip is given in prose or verse many commentaries that are interesting and instructive. It is certainly one of the most useful and well-appointed desk calendars that is issued, copies of which can be obtained by sending five two-cent stamps to the Calendar Department, Pope Manufacturing Company, Hartford, Conn.

W. B. Saunders, publisher, 925 Walnut street, Philadelphia, sent out at the close of the year 1896 a bulletin of his new publications for 1896-97. Accompanying it is a preliminary announcement and a few sample pages of Dr. Gould's new book on Anomalies and Curiosities of Medicine. It can be seen by the announcement that years of labor have been expended in collecting the material. It is stated that the authors have spent several years in the libraries of America and Europe in gathering the material for the book. It is announced that this book will be one of the most important works of the coming season and one sure to attract widespread attention.

